# Genetic and Physio-Biochemical Characterization of a Novel Premature Senescence Leaf Mutant in Rice (*Oryza sativa* L.)

**DOI:** 10.3390/ijms19082339

**Published:** 2018-08-09

**Authors:** Yan He, Zhihong Zhang, Liangjian Li, Shaoqing Tang, Jian-Li Wu

**Affiliations:** State Key Laboratory of Rice Biology, China National Rice Research Institute, Hangzhou 310006, China; chinayanyan@163.com (Y.H.); 13650950747@163.com (Z.Z.); 15958018630@163.com (L.L.); sqtang@126.com (S.T.)

**Keywords:** rice, premature senescence, chlorophyll, abscisic acid, senescence-associated gene

## Abstract

Premature senescence greatly affects the yield production and the grain quality in plants, although the molecular mechanisms are largely unknown. Here, we identified a novel rice *premature senescence leaf 85* (*psl85*) mutant from ethyl methane sulfonate (EMS) mutagenesis of cultivar Zhongjian100 (the wild-type, WT). The *psl85* mutant presented a distinct dwarfism and premature senescence leaf phenotype, starting from the seedling stage to the mature stage, with decreasing level of chlorophyll and degradation of chloroplast, declined photosynthetic capacity, increased content of malonaldehyde (MDA), upregulated expression of senescence-associated genes, and disrupted reactive oxygen species (ROS) scavenging system. Moreover, endogenous abscisic acid (ABA) level was significantly increased in *psl85* at the late aging phase, and the detached leaves of *psl85* showed more rapid chlorophyll deterioration than that of WT under ABA treatment, indicating that *PSL85* was involved in ABA-induced leaf senescence. Genetic analysis revealed that the premature senescence leaf phenotype was controlled by a single recessive nuclear gene which was finally mapped in a 47 kb region on the short arm of chromosome 7, covering eight candidate open reading frames (ORFs). No similar genes controlling a premature senescence leaf phenotype have been identified in the region, and cloning and functional analysis of the gene is currently underway.

## 1. Introduction

Leaf senescence occurs in the final stage of plant development, with a series of complex physiological and biochemical alterations. As a form of programmed cell death (PCD), leaf senescence is a genetically controlled system of self-destruction during which leaf cells experience massive alterations in metabolic processes, cellular structures, and gene expressions [1,2]. The most obvious visible sign of senescence is the leaf yellowing phenotype, which is caused by preferential breakdown of chlorophyll and chloroplasts, and accompanied by hydrolysis of macromolecules, such as proteins, lipids, and nucleic acids, and degeneration of mitochondria and nuclei [3,4]. During this senescence process, nutrients, such as nitrogen and phosphorus, are reallocated to the younger developing tissues to increase reproductive success [5]. Thus, plant senescence is not a purely negative or passive process.

As the specialized photosynthesis organ, leaf tissues play an important role in productivities of carbohydrate and energy. The transition from carbon assimilation to nutrient remobilization during the leaf aging process involves the chloroplast disassembly, and as a result, the disruption of chloroplast structure results in rapid breakdown of chlorophyll and negatively influences the photosynthetic efficiency in aging leaves [6,7]. The thylakoid membrane in chloroplasts is the main site where light energy is converted into metabolically usable forms by photosynthesis, and the photosystem (PS) I, PSII, cytochrome (cyt) *b6f*, light-harvesting complex (LHC) I, LHCII, and ATPase complexes are concentrated in the stacked and unstacked grana thylakoid membrane [8]. In the process of senescence, chlorophyll is removed from the thylakoid membrane and converted to colorless intermediate products by a multistep pathway, and which are then subsequently stored in vacuoles [9]. Additionally, the levels of many photosystem proteins, such as Lhca1, Lhcb1, and PsaA are degraded with the onset of leaf senescence, which might directly lead to a lower photosynthetic capacity [10,11].

Reactive oxygen species (ROS) accumulation and DNA degradation have been reported to be involved in promoting premature senescence [12,13]. Thus, ROS scavenging enzymes, such as superoxide dismutase (SOD), peroxidase (POD), and catalase (CAT), play significant regulatory roles in leaf senescence. Malonaldehyde (MDA) and membrane ion leakage, two indicators of cell membrane damage, are generally present in higher levels in premature senescence rice mutants, compared to their wild types [10,14,15]. In previous studies, some premature senescence leaf mutants, like *psl50*, *psl117* [6], and *lts1* [16], it was reported that numerous DNA fragmentation events and cell death occurred in the leaves with the initiation and progression of premature senescence phenotype.

In the onset and progression of senescence process, plants also integrate multiple internal and external signals to respond to various types of endogenous and exogenous aging-effected factors through intricate regulatory pathways [17,18]. Abscisic acid (ABA) plays an important role in environmental stress responses and, thus, leaf longevity [5]. Consistently, ABA is also thought to facilitate leaf aging and abscission, and both altered expression levels of ABA metabolism-related genes and increased levels of endogenous ABA have been detected in the leaves that undergo senescence [18,19]. Furthermore, a large number of differentially expressed genes (DEGs) have been identified in leaf senescence plants, induced by biotic or/and abiotic stresses, and exogenous ABA treatment has been reported to cause the upregulation of several senescence-associated genes (SAGs) and chlorophyll degradation-related genes (CDGs) known to accelerate leaf senescence, such as *Osh36*, *OsI57*, *stay-green* (*SGR*), and *red chlorophyll catabolite reductase 1* (*RCCR1*) [20,21,22]. In addition, darkness is a well-known powerful external stimulus to accelerate leaf senescence [23]. As demonstrated by previous studies, dark-induced leaf senescence was accelerated in the rice premature senescence leaf mutants *rls1* [24], *ps1-D* [20] and *osphyB-2* [25].

Premature leaf senescence has a great impact on the crop yield and grain quality, while the underlying molecular mechanism of senescence is still poorly understood [26]. Here, a novel rice premature senescence leaf mutant, tentatively named *psl85*, obtained from an ethyl methane sulfonate (EMS) mutant bank of rice cultivar Zhongjian100, was identified. We investigated the physiological and biochemical properties which could contribute to the yellowish leaf phenotype and impaired performance of agronomic traits in *psl85*. In contrast to WT, *psl85* showed more rapid chlorophyll degradation both under field conditions and after ABA treatment. Furthermore, the altered expression of genes related to SAGs, CDGs, and ABA metabolism, in *psl85*, indicated the presence of a link between *PSL85* and ABA signaling pathway. Our results would facilitate the study on the molecular mechanism of premature leaf senescence in rice, and also provide a foundation for isolation and functional analysis of *PSL85*.

## 2. Results

### 2.1. Phenotypic Performance of psl85

**Figure 1:**
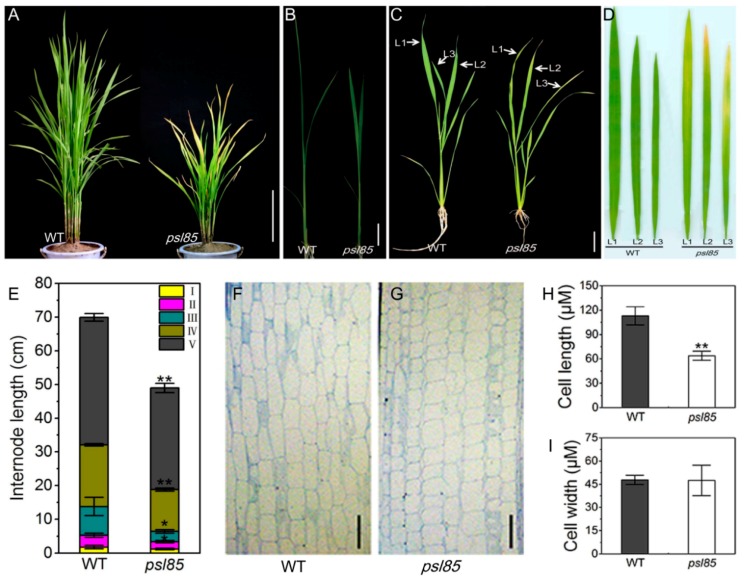
Phenotypes of wild type (WT) and *psl85*. (**A**) WT and *psl85* at the tillering stage. (**B**) WT and *psl85* seedlings at DAG15. (**C**) WT and *psl85* at DAG30. L1, L2, and L3 indicate the top three leaves of WT and *psl85*. (**D**) Enlarged views of top three leaves of WT and *psl85* in (**C**). (**E**) Internode length of the main stem at the mature stage in WT and *psl85.* (**F**,**G**) Longitudinal section of the third internode of WT (**F**) and *psl85* (**G**) at the mature stage. Scale bar = 100 µm. (**H**,**I**) Longitudinal cell length (**H**) and cell width (**I**) of WT and *psl85.* Scale bar = 20 cm in (**A**) and scale bar = 2 cm in (**B**,**C**). Values are means ± SD (*n* = 3); ** indicates significance at *p* ≤ 0.01 and * indicates significance at *p* ≤ 0.05 by Student’s *t* test.

The *psl85* mutant exhibited a premature senescence leaf phenotype (yellowish leaves) and dwarfism under the field and greenhouse conditions in Hangzhou, Zhejiang, China, and Lingshui, Hainan, China. The leaf senescence phenotype appeared in *psl85* about 25 days after germination (DAG25) and lasted until the mature stage under field conditions, compared with the wild type (WT) Zhongjian 100, (Figure 1A,C). In *psl85*, the leaf yellowing phenotype was initiated from the leaf tip and spread downward to the bottom part of leaves (Figure 1D). In addition to the distinct leaf yellowing phenotype, *psl85* mutant showed a decreased plant height with shortened internodes at the seedling, tillering, and mature stages compared to WT (Figure 1A,B,E). Other major agronomic traits, including the panicle length, number of filled grains per plant, and seed-setting rate, were all remarkably reduced in *psl85* (Table S1), indicating that the premature leaf senescence in *psl85* would impose a negative effect on the plant yield. To investigate the direct reason for dwarfism in *psl85*, we performed paraffin sectioning to compare the cell size between WT and *psl85* (Figure 1F,G). The results showed that the cell length was significantly reduced in the mutant, while the cell width was similar between *psl85* and WT (Figure 1H,I), which indicated that the dwarf phenotype of *psl85* was directly resulted from the reduced cell length of the stems.

### 2.2. Alterations of Chlorophyll Contents, Chloroplast Ultrastructure, and Photosynthetic Parameters

**Figure 2:**
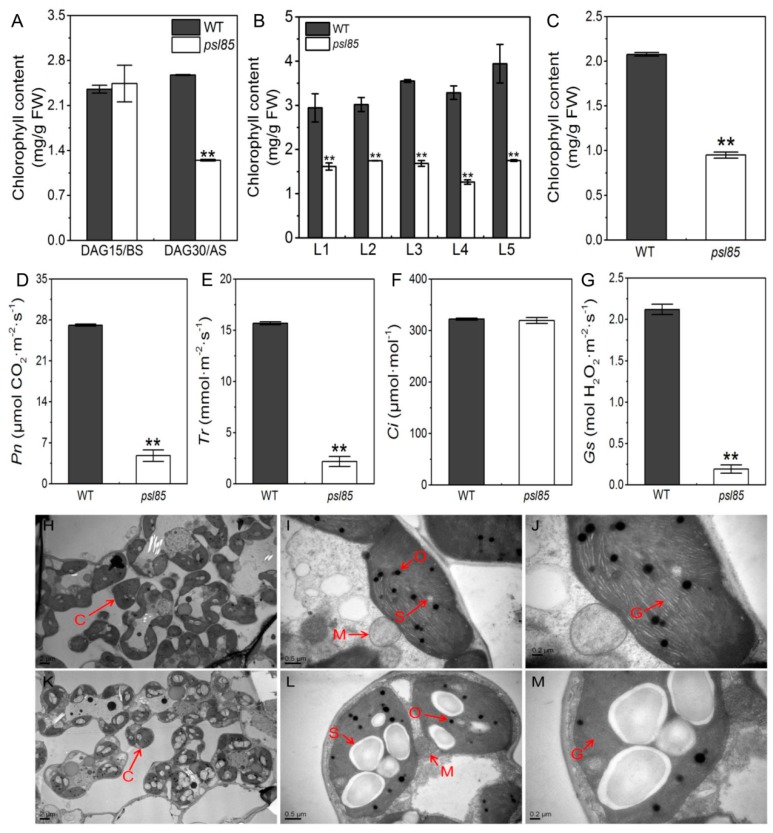
Chlorophyll contents, chloroplast structures, and photosynthetic parameters in WT and *psl85*. (**A**) Chlorophyll contents of WT and *psl85* at DAG15 and DAG30. BS, beginning of senescence. AS, after senescence. (**B**) Chlorophyll contents of the upper five leaves at DAG60. (**C**) Chlorophyll contents of flag leaves at the heading stage. (**D**–**G**) Photosynthetic parameters of flag leaves at the heading stage. Net photosynthetic rate (*Pn*), transpiration rate (*Tr*) intercellular CO_2_ concentration (*Ci*) and stomatal conductance (*Gs*). (**H**–**J**) Ultrastructure of chloroplast in WT. (**K**–**M**) Ultrastructure of chloroplast in *psl85*. C, chloroplast; O, osmiophilic granule; S, starch granule; G, grana thylakoid. Values are means ± SD (*n* = 3); ** indicates significance at *p* ≤ 0.01 by Student’s *t* test.

To examine whether the yellowing phenotype was associated with the chlorophyll level, we measured the chlorophyll contents of *psl85* mutant at DAG15 before senescence, DAG30, and DAG60 after senescence. The results showed that the chlorophyll levels were similar between WT and *psl85* at DAG15, while there was a significant reduction of chlorophyll levels in *psl85* at DAG30, and the upper five leaves at DAG60 compared with WT (Figure 2A,B). Furthermore, a significantly decreased chlorophyll content of the functional flag leaves was also observed in *psl85* at the heading stage (Figure 2C).

Premature leaf senescence is often coupled with defective photosynthesis in plants [27]. To test this possibility, we examined the photosynthetic parameters of flag leaves in WT and *psl85* at the heading stage. The results showed that the values of net photosynthetic rate (*Pn*), transpiration rate (*Tr*), and stomatal conductance (*Gs*) were significantly lower in *psl85* than those of WT (Figure 2D,E,G), while the values of intercellular CO_2_ concentration (*Ci*) were similar between WT and *psl85* (Figure 2F). These results strongly demonstrated that the photosynthetic capacity was impaired in *psl85*.

To explore the cause of lower chlorophyll level in *psl85* during senescence, we compared the chloroplast ultrastructure of mesophyll cells in fully expanded flag leaves in *psl85* and WT at the heading stage, by transmission electron microscopy (TEM). In comparison to WT, the lower number of chloroplast and starch granule were observed, and the size of starch granule was much larger in mesophyll cells of *psl85* (Figure 2H,I,K,L). Furthermore, degenerated thylakoid membrane and a decreased number of grana thylakoid were observed in *psl85* when compared to WT (Figure 2J,M). These features indicated that the mutation responsible for the premature senescence leaf phenotype of *psl85* could result in chloroplast dysplasia.

### 2.3. Impaired Photosynthesis System in psl85 Mutant

**Figure 3:**
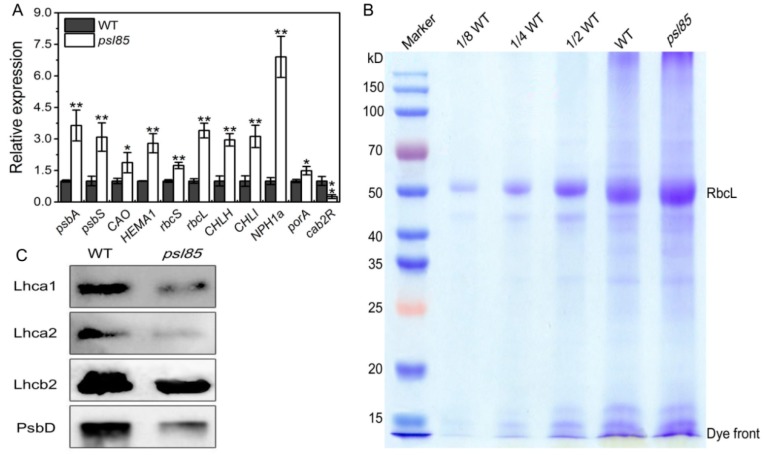
Analysis of photosynthesis-related gene expression and protein levels. (**A**) Expression profile of photosynthesis-related genes in flag leaves of WT and *psl85* at the heading stage. (**B**) Coomassie brilliant blue staining of total proteins. Total proteins extracted from flag leaves of WT and *psl85* at the heading stage were resolved by polyacrylamide sodium dodecyl sulfate-polyacrylamide gel electrophoresis (SDS-PAGE) and quantified by bicinchoninic acid (BCA), and the major band of the large subunit of ribulose bisphosphate carboxylase (RbcL) is indicated. (**C**) Immunoblot analysis of Lhca1, Lhca2, Lhcb2, and PsbD in the total proteins extracted from flag leaves of WT and *psl85* at the heading stage. The immunoblot experiment was repeated three times, independently, and a similar result was obtained each time. Values are means ± SD (*n* = 3); ** indicates significance at *p* ≤ 0.01 and * indicates significance at *p* ≤ 0.05 by Student’s *t* test.

To clarify the reasons for the defective photosynthesis in *psl85* mutant, we further performed a qRT-PCR analysis on the expression of a set of photosynthesis-related genes in flag leaves from WT and *psl85* at the heading stage. Unexpectedly, 10 out of 11 photosynthesis-related genes exhibited significant higher expression levels in *psl85* in contrast to WT, while the expression level of *cab2R* was significantly decreased in *psl85*, compared to that of WT (Figure 3A), which indicated that the mutation of *PSL85* might cause a positive regulation to the multiple genes tested. We next analyzed the protein profile to further investigate the stability of photosynthetic proteins in *psl85*. The total proteins from flag leaves of WT and *psl85* were quantified to a similar concentration by using a bicinchoninic acid (BCA) method, then tested by Coomassie brilliant blue staining (Figure 3B). Additionally, an immunoblot experiment was carried out to analyze the levels of proteins that form the subunits of photosynthetic complexes, including PSI type I chlorophyll a/b binding protein (Lhca1), PSI type II chlorophyll a/b binding protein (Lhca2), LHCII type II chlorophyll a/b binding protein (Lhcb2), and D2 protein of PSII (PsbD). The results showed that the protein levels were all apparently reduced in *psl85* compared with WT (Figure 3C). Taken together, the significant reduction of the representative subunits of photosynthetic complexes revealed that the thylakoid membrane was very likely disrupted, and resulted in the poor performance of photosynthesis in *psl85*.

### 2.4. Alterations of Physio-Biochemical Indicators Related to Senescence at Different Growth Stages

**Figure 4:**
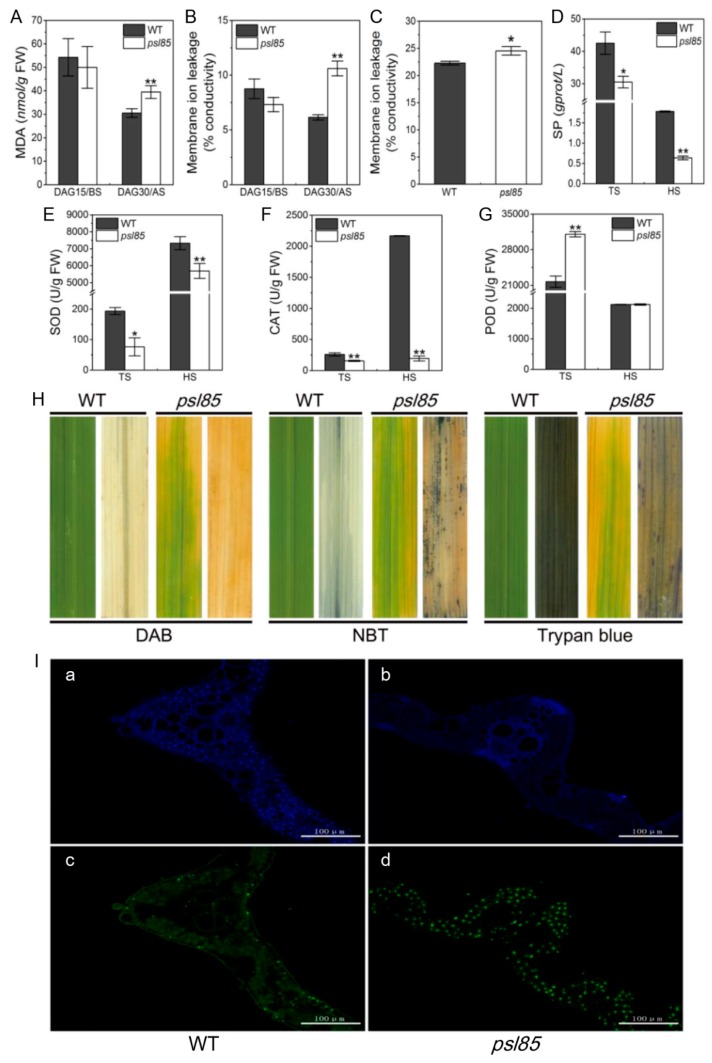
Senescence-related parameters, and histochemical and TUNEL assays of WT and *psl85*. (**A**,**B**) Malonaldehyde (MDA) contents (**A**) and membrane ion leakage rates (**B**) of WT and *psl85* at DAG15 and DAG30. BS, beginning of senescence. AS, after senescence. (**C**) Membrane ion leakage rates of WT and *psl85* at the heading stage. (**D**–**G**) Soluble protein (SP) content (**D**), superoxide dismutase (SOD) activity (**E**), catalase (CAT) activity (**F**), and peroxidase (POD) activity (**G**) of WT and *psl85* at the tillering stage and heading stage. TS, tillering stage; HS, heading stage. The top second leaves at the tillering stage and the fully expanded flag leaves at the heading stage were used for analysis in all experiments. Values are means ± SD (*n* = 3); ** indicates significance at *p* ≤ 0.01 and * indicates significance at *p* ≤ 0.05 by Student’s *t* test. (**H**) DAB assay, NBT assay, and trypan blue staining in WT and *psl85* at the tillering stage. (**I**) TUNEL assay of WT and *psl85* at the tillering stage. Blue signal represents 4′,6-diamino-phenylindole (DAPI) staining; green color represents positive result. **a** and **b** are negative controls for **c** and **d**, respectively.

The cell membrane damage of leaves in WT and *psl85* before senescence (DAG15) and after senescence (DAG30) was estimated by measuring the malonaldehyde (MDA) content and ion leakage, two cell membrane damage indicators. The results showed that the MDA content and the value of membrane ion leakage were significantly increased in *psl85* at DAG30, compare to those of WT at DAG30 and the heading stage, however, both of them were not remarkably different between WT and *psl85* at DAG15 (Figure 4A–C). We then examined the soluble protein levels, and found that *psl85* exhibited a more rapid degradation of soluble proteins than WT, both at the tillering stage and heading stage (Figure 4D). These results indicated that the premature senescence leaf phenotype in *psl85* was accompanied by the degradation of proteins and the damage of cell membranes.

The reactive oxygen species (ROS) is well known to directly kill cells, and is closely associated with senescence in plants. To further explore the causes of premature leaf senescence in *psl85*, we further examined the activities of ROS scavenging enzymes containing superoxide dismutase (SOD), catalase (CAT) and peroxidase (POD). In comparison with WT, the SOD and CAT activities were significantly decreased in *psl85* both at the tillering and heading stages (Figure 4E,F). By contrast, the activity of POD was apparently higher in *psl85* than that of WT at the tillering stage, while there was no obvious difference between WT and *psl85* at the heading stage (Figure 4G). POD has been reported to catalyze the oxidation of cinnamyl alcohols for lignin condensation in plants, and the activities of POD are generally higher in aging plant tissues than those of young plant tissues [28]. Our results may indicate a higher degree of lignification in *psl85* during the premature senescence process, although further evidence is required. All these results clearly demonstrated that the degradation of soluble proteins, cell membrane damage, and disordered ROS scavenging capacity would accelerate the leaf senescence in *psl85*.

To detect whether ROS accumulation and cell death indeed occurred in *psl85*, the same part of top second leaves from WT and *psl85* at the tillering stage were stained with trypan blue, 3,3′-diaminobenzidine (DAB), and nitrotetrazolium blue chloride (NBT), respectively. The results showed that there were numerous blue formazan precipitates in *psl85* leaves, while almost no blue formazan precipitates were observed in WT, indicating that O_2_^−^ accumulation did occur in *psl85* (middle, Figure 4H). Similarly, higher numbers of blue spots were observed in *psl85* leaves than those of WT after trypan blue staining, indicating the presence of cell death in *psl85* (Right, Figure 4H). As for H_2_O_2_ accumulation, a few tiny brown precipitates were detected in *psl85* leaves after DAB staining, while almost no such signal was detected in WT leaves (Left, Figure 4H). The results demonstrated that the ROS accumulation and cell death did occur upon the progression of premature leaf senescence in *psl85*.

We further performed a terminal deoxyribonucleotidyl transferase-mediated dUTP nick-end labeling (TUNEL) assay on the top second leaves at the tillering stage for confirming cell death at the DNA molecular level. The same leaf sections were simultaneously stained with 4′,6-diamino-phenylindole (DAPI) to reveal the nuclei (blue). The results showed that a few nuclei (green) were TUNEL positive in WT, whereas numerous nuclei were TUNEL positive in *psl85* (Figure 4I). Our results confirmed that the mutation of *PSL85* would induce large scale DNA damage, leading to cell death, as shown by trypan blue staining.

### 2.5. Elevated Expression of Chlorophyll Metabolism and Senescence-Associated Genes by qRT-PCR

**Figure 5:**
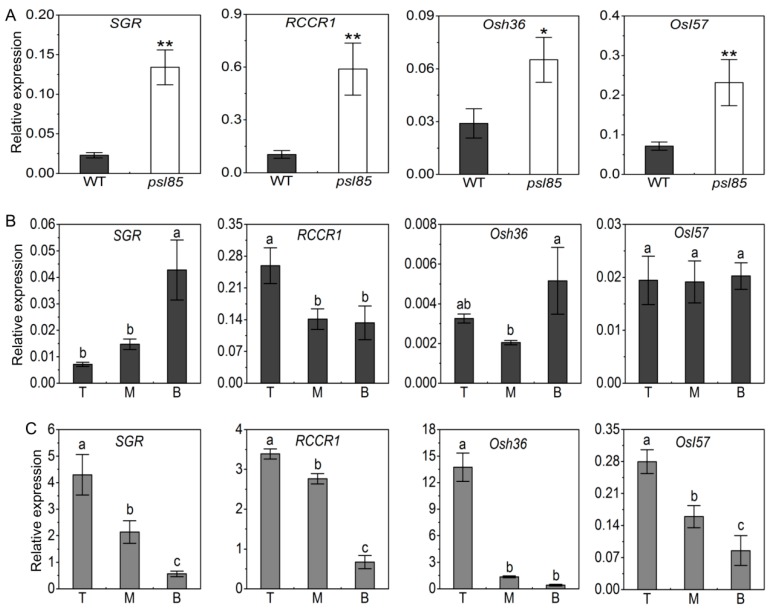
Expression of chlorophyll metabolism and senescence-associated genes. (**A**) Expression of CDGs (*SGR* and *RCCR1*) and SAGs (*Osh36* and *OsI57*) in flag leaves of WT and *psl85*. Values are means ± SD (*n* = 3), ** indicates significance at *p* ≤ 0.01 and * indicates significance at *p* ≤ 0.05 by Student’s *t* test. (**B**,**C**) Expression of CDGs (*SGR* and *RCCR1*) and SAGs (*Osh36* and *OsI57*) in different parts of fully expanded leaves in WT (**B**) and *psl85* (**C**) at the tillering stage. T, top portion leaves; M, middle portion leaves; B, bottom portion leaves. Values are means ± SD (*n* = 3), different letters indicate significance at *p* ≤ 0.05 by Duncan’s test.

To examine whether the expression of chlorophyll metabolism and senescence-associated genes had been altered, we further investigated the expression of two chlorophyll degradation-related genes (CDGs), *stay-green* (*SGR*) and *red chlorophyll catabolite reductase 1* (*RCCR1*), and two other senescence-associated genes (SAGs), *Osh36* and *OsI57* [20], in the flag leaves of WT and *psl85*. Our results indicated that both of the two CDGs (*SGR* and *RCCR1*) and two SAGs (*Osh36* and *OsI57*) were significantly upregulated in *psl85* compared with WT (Figure 5A). In addition, the level of CDG and SAG transcripts decreased gradually from the top to the bottom of fully expanded leaves, consistent with the onset of senescence progression in *psl85* at the tillering stage (Figure 5C), while no such a distinct tendency of expression was observed in different parts of fully expand leaves in WT (Figure 5B). Overall, the data suggested that the accelerated leaf senescence process in *psl85* was likely resulted from the elevated expression of CDGs and SAGs.

### 2.6. Induction of Synchronous Senescence by Darkness and ABA

**Figure 6:**
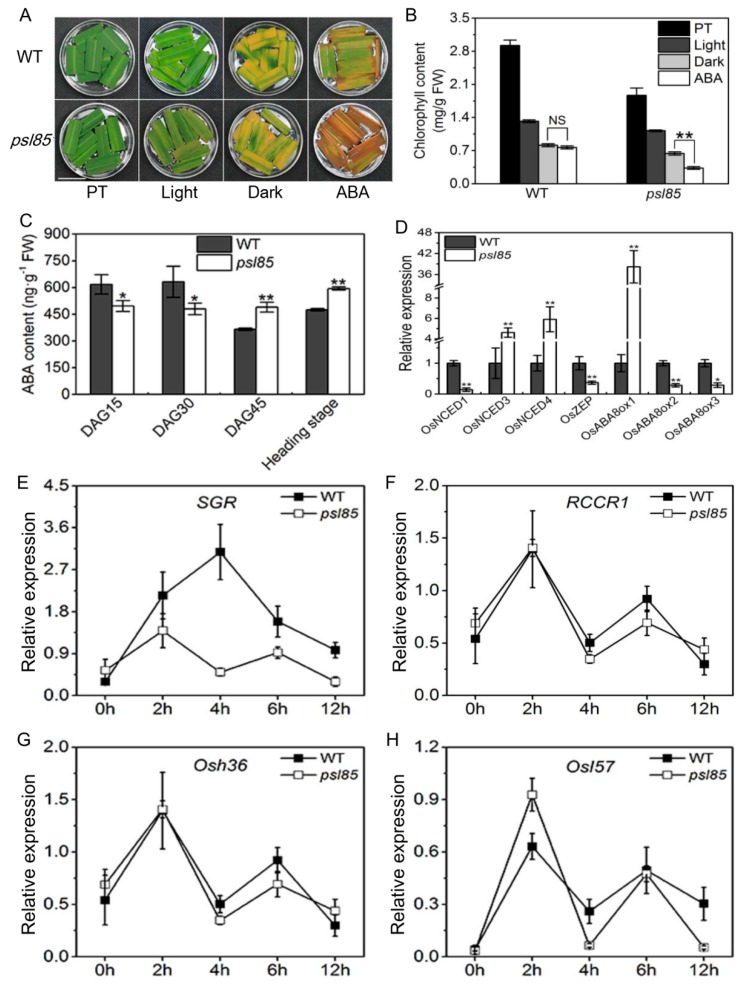
Analysis of darkness- and ABA-induced leaf senescence. (**A**) Detached fully expanded leaves from WT and *psl85* at the tillering stage were incubated under continuous light (H_2_O), darkness (H_2_O), and 50 µM ABA (continuous light) at 28 °C for 5 days. PT, pretreatment; Scale bar = 2 cm. (**B**) Chlorophyll content of the detached leaves shown in A. Values are means ± SD (*n* = 3); NS, no significance; ** indicates significance at *p* ≤ 0.01 by Student’s *t* test. (**C**) ABA content in WT and *psl85* at different growth stages. (**D**) Expression of ABA-related genes in flag leaves from WT and *psl85* at the heading stage. ** indicates significance at *p* ≤ 0.01 and * indicates significance at *p* ≤ 0.05 by Student’s *t* test. (**E**–**H**) Kinetic expression of two CDGs and two SAGs after treatment with 50 µm ABA. Values are means ± SD (*n* = 3).

Leaf senescence is a genetically controlled developmental process that can be modulated by a variety of phytohormones and environmental factors, and it is well known that both abscisic acid (ABA) and darkness could induce leaf senescence in rice [20,22]. To confirm whether the *PSL85* mutation would accelerate leaf senescence in *psl85* under darkness and ABA treatment, the detached leaves from *psl85* and WT were subjected to 5-day darkness and ABA treatments, while the light condition was used as control. The results showed that the mutation of *PSL85* significantly accelerated ABA-induced leaf senescence, rather than dark-induced leaf senescence (Figure 6A,B). Subsequently, we examined the ABA levels of WT and *psl85* at different growth stages, and it showed that the ABA contents were significantly lower in *psl85* than those of WT at DAG15 and DAG30, whereas at DAG45 and the heading stage, the ABA contents were apparently higher in *psl85* than those of WT (Figure 6C), indicating that endogenous ABA may play an important role on the progression of rapid senescence of *psl85*. Further examination of the expression levels of a number of key ABA biosynthesis genes (*OsNCED1*, *OsNCED3*, *OsNCED4*, and *OsZEP*), and ABA-inactivation genes (*OsABA8ox1*, *OsABA8ox2*, and *OsABA8ox3*) indicated that *OsNCED3*, *OsNCED4*, and *OsABA8ox1* were significantly upregulated, while others were significantly downregulated in *psl85* compared to WT (Figure 6D). All these results implied that the normal ABA metabolism pathway was disordered in *psl85*, due to the mutation of *PSL85*.

We also investigated the kinetic expression of two CDGs and two SAGs in WT and *psl85* after ABA treatment. The dynamic changes of *RCCR1*, *Osh36*, and *OsI57* expression exhibited a similar trend at different time points both in *psl85* and WT after ABA treatment, while a huge difference in *SGR* expression, especially at 4h time point, was observed between WT and *psl85* after ABA treatment (Figure 6E–H). This deviation suggested that the *PSL85* might provide a link between *SGR* and ABA-mediated leaf senescence. Taken together, it seems that the mutation causes premature leaf senescence that is associated with ABA signaling pathway.

### 2.7. Genetic Analysis and Mapping of PSL85

To determine the genetic control of the premature senescence leaf phenotype of *psl85*, we crossed *psl85* with the japonica cultivar Moroberekan. All the F_1_ plants from the cross *psl85*/Moroberekan showed the normal green phenotype similar to WT. In the F_2_ segregation population, 1,128 plants displayed the normal green phenotype, and 352 plants exhibited the yellowing phenotype similar to *psl85*, fitting a 3:1 ratio (χ^2^ = 1.17 < χ^2^_0.05_ = 3.84). The results suggested that the mutation was controlled by a single recessive nuclear gene.

**Figure 7:**
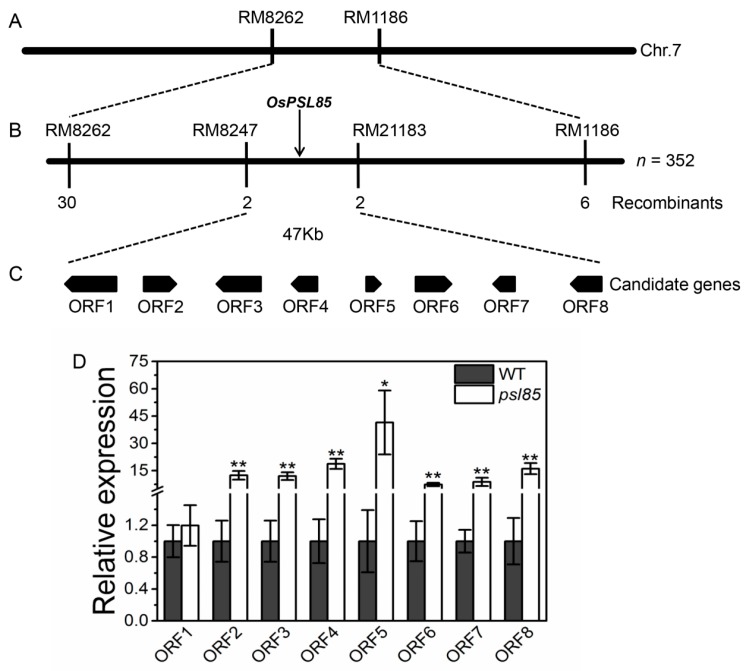
Fine mapping of the *PSL85* gene and expression analysis of candidate genes. (**A**–**C**) Fine mapping of the *PSL85* gene. (**D**) Expression level analysis of candidate genes in flag leaves from WT and *psl85* at the heading stage. Values are means ± SD (*n* = 3); ** indicates significance at *p* ≤ 0.01 and * indicates significance at *p* ≤ 0.05 by Student’s *t* test.

We further performed map-based cloning to locate the *PSL85* gene by using the *psl85* type F_2_ individuals derived from the same cross. A total of 362 polymorphic simple sequence repeat (SSR) markers between two parents were used for linkage analysis in two bulked segregant analysis (BSA) DNA pools. The results showed that 3 SSR markers (RM5752, RM8262, and RM1186) on the short arm of chromosome 7 were co-segregated with the mutation (Figure 7A). Subsequently, fine mapping was conducted by genotyping all 352 F_2_ individuals with the *psl85* phenotype, and the *PSL85* gene was finally delimited to a 47 kb region between markers RM8247 and RM21183 (Figure 7B).

According to the Rice Genome Annotation Project (http://rice.plantbiology.msu.edu/), 8 putative open reading frames (ORFs) were identified in the target region (Figure 7C and Table 1). Subsequently, the expression levels of these eight ORFs were compared between WT and *psl85* by qRT-PCR. Unexpectedly, 7 out of 8 OFRs exhibited significantly higher expression levels in *psl85* than those of WT, except the ORF1 expression level, which was similar to that of WT (Figure 7D). Therefore, further study is needed to identify the candidate *PSL85* gene responsible for the premature senescence leaf phenotype.

## 3. Discussion

Leaf senescence is a complex and highly fine-tuned process, which is involved in chloroplast disintegration, macromolecule catabolism, nuclei degeneration, and SAG regulation. Despite that leaf senescence is an important biological step in the whole life cycle of a plant, the molecular mechanism underlying senescence still remains largely unknown, due to its extreme complexity. In our work, the leaf senescence process was accelerated remarkably in *psl85* from the seedling stage to grain-filling stage, with enormous changes of physiological and biochemical properties, such as declined photosynthetic capacity, increased MDA content, and disrupted ROS scavenging system, which might be responsible for the premature leaf senescence phenotype, and consequently resulted in the poorer performance of major agronomic traits in *psl85*.

As a genetically controlled process of cell suicide, programmed cell death (PCD) is strictly regulated and important for plant growth, development, and biotic/abiotic stress responses [29]. The triggering of PCD is generally accompanied by the increase of various ROS (H_2_O_2_, O_2_^−^ etc.) levels, and it has been widely accepted that upregulated activities of ROS scavenging enzymes, like SOD, POD and CAT, are closely involved in the detoxification of ROS [30,31,32]. Here, we observed that PCD was misgoverned in *psl85* leaves at the tillering stage, in contrast to WT (Figure 4H,I). Expectedly, the SOD and CAT activities presented at lower levels in leaves of *psl85* at different stages (Figure 4E,F). Meanwhile, the role of POD activity in leaf senescence remains controversial, as both up-/downregulated POD activities have been reported in different rice premature leaf senescence mutants [6,14,33]. For *psl85*, the activity of POD was higher at the tillering stage, but similar at the heading stages, compared to WT (Figure 4G). Nevertheless, all these features provided evidence for the involvement of *PSL85* in the ROS–PCD pathway-mediated leaf senescence.

Effective and efficient leaf photosynthesis is indispensable for growth, development, and high yielding in plants [34,35]. Although most of the tested photosynthesis-related genes (10/11) were upregulated in *psl85*, the degradation of photosynthetic proteins was accelerated in *psl85*, leading to the dramatic decline of photosynthesis in *psl85* compared to WT (Figure 2C–F and Figure 3). Intriguingly, the *Ci* values were similar between WT and *psl85*, and we speculated that more CO_2_ was absorbed from external environment through stomata in WT because of its higher *Gs*, however, the higher *Pn* might cause a higher CO_2_ consumption in WT than in *psl85*, which consequently resulted in the relatively similar level of *Ci* between WT and *psl85*. Nevertheless, the structural degeneration of thylakoids in chloroplasts and rapid loss of chlorophyll during the premature senescence process would be the main causes of the lower photosynthesis capacity in *psl85*.

Although ABA and darkness are tightly involved in triggering of plant senescence [20,36], the mechanism of ABA- and darkness-promoted senescence remains unclear. In *psl85*, we found that the response of detached leaves to ABA was more sensitive than that to darkness (Figure 6A,B). The endogenous ABA level is not only regulated by its biosynthesis, but also by its catabolism, and the cleavage of 9-*cis* epoxycarotenoids to xanthoxin is a key regulatory step in ABA biosynthesis catalyzed by the enzyme 9-*cis*-epoxycarotenoid dioxygenase (NCED), while the generation of phaseic acid (PA) from ABA catabolism begins with the hydroxylation of the 8′ position by ABA 8′-hydroxylase (ABA8ox) [37,38,39,40]. Compared with WT, a higher endogenous level of ABA was detected in *psl85* at the later phase of aging (Figure 6C), with the upregulation of two ABA biosynthetic genes (*OsNCED3* and *OsNCED4*) and one ABA degradation gene (*OsABA8ox1*), and the downregulation of two ABA biosynthetic genes (*OsNCED1* and *OsZEP*) and two ABA degradation gene (*OsABA8ox2* and *OsABA8ox3*) (Figure 6D). Notably, the ABA degradation gene, *OsABA8ox1*, was upregulated by approximately 40 times in *psl85* than that of WT at the heading stage, while the higher endogenous ABA level in *psl85* could possibly be contributed to by the two highly upregulated ABA biosynthetic genes (*OsNCED3* and *OsNCED4*) (Figure 6C,D). Under ABA treatment, although *psl85* showed a quite similar pattern of kinetic expression among different types of senescence-marker genes (SAGs and CDGs), however, the expression pattern of *SGR* was significantly different between the mutant and WT. This differential expression pattern requires further characterization. Based on the results, we speculated that *PSL85* may act as a link between *SGR*-mediated leaf senescence and ABA signaling pathway.

The mutation of *PSL85* responsible for *psl85* phenotype was delimited to a 47 kb physical region covering 8 ORFs on the short arm of chromosome 7. So far, no evidence shows that these eight ORFs are associated with premature leaf senescence, thus *psl85* is likely a novel rice premature senescence leaf mutant. It has been shown that the mutation of a gene would result in alteration of its expression level [41,42]. In order to identify the target ORF, an expression analysis of candidate ORFs was performed in WT and *psl85*. Unfortunately, none of them is considered as a candidate gene, because of the similar expression level between the mutant and WT, or highly elevated levels of expression in the mutant (Figure 7D). Hence, other strategies, such as DNA sequencing of the ORFs, including CDS and promoters, should be carried out to identify the target gene. Previous studies have demonstrated that two CDGs (*SGR* and *RCCR1*) and two SAGs (*Osh36* and *OsI57*) were highly expressed in senescing tissues of rice, and involved in the aging process [20,43,44,45]. In the present study, the upregulation of these CDGs and SAGs were detected in the flag leaves of *psl85* at the heading stage, and their expression levels decreased gradually, from the top part to the basal part of a fully expanded leaf (Figure 5A,C). These results implied that *PSL85* accelerated leaf senescence in a SAG- and CDGs-dependent manner.

In conclusion, our results provide the foundation for cloning and functional analysis of the *PSL85* gene, an ideal marker for the senescence process in rice, and would facilitate the elucidation of PSL85-mediated molecular mechanism of senescence.

## 4. Materials and Methods

### 4.1. Plant Materials and Growth Conditions

The *psl85* mutant was obtained from an ethane methyl sulfonate (EMS)-induced indica rice Zhongjian100 (the wild-type, WT) mutant bank. It has been selfed for more than 10 generations, and the premature senescence leaf phenotype has been stably expressed under the field and greenhouse conditions in Fuyang, Hangzhou, Zhejiang, China and Lingshui, Hainan, China. *psl85* was crossed to the japonica rice Moroberekan, all the F_1_ plants and F_2_ individuals were grown in the paddy field at the China National Rice Research Institute (CNRRI) for genetic analysis and gene mapping. For the expression analysis of CDGs and SAGs after ABA induction, WT and *psl85* plants were hydroponically cultured with Hoagland medium after germination in the growth chamber (MLR-352H-PC, Panasonic, Osaka, Japan) for 10 days at 28 °C and with a 14 h light/24 °C, 10 h dark cycle. Whole 10-day-old WT and *psl85* plants were immersed in Hoagland medium containing 50 μM ABA for 2, 4, 6, and 12 h. Three WT and *psl85* plants were collected at each time point for RNA isolation.

### 4.2. Histochemical Analysis

The uppermost leaves of WT and *psl85* at the tillering stage in the paddy field at CNRRI were collected, and the leaf blades were cut into ~5 cm pieces for detection of H_2_O_2_ and O_2_^−^ accumulation by 3,3-diaminobenzidine (DAB) staining and nitrotetrazolium blue chloride (NBT) staining, respectively, following the methods described by Wang et al. [46]. The above leaf samples were also used for cell death assay by trypan blue staining, following the method described by Yin et al. [47]. The pictures were recorded using a HP ScanJet G4010 scanner (HP, Shanghai, China).

### 4.3. Chlorophyll Content and Membrane Ion Leakage Determination

Chlorophyll was extracted from 25 mg of fresh leaf tissues, and its content was determined by measuring the absorbance at 652 nm using a SpectraMax i3x Multi-Mode Microplate Reader (MOLECULAR DEVICES, Sunnyvate, CA, USA) as described previously [4]. Leaves were scissor-cut into ~5 mm pieces and 50 mg was weighed out precisely, then placed into 20 mL deionized water in a tube, followed by vacuum treatment for 10 min, and incubated in a shaker with 100 rpm at 28 °C for 30 min. The membrane ion leakage (value A) was measured using a DDS-307A conductivity meter (LeiCi, Hangzhou, China). Then, the samples were incubated in boiling water for 5 min to thoroughly release electrolytes. After the samples were cooled down to the room temperature, the final membrane ion leakage (value B) was measured. The value of membrane ion leakage was calculated by the formula A/B × 100%. The means from three measurements in both experiments were used for analysis.

### 4.4. Senescence-Related and Photosynthesis Parameter Measurement

The leaf blades from WT and *psl85* at different growth stages from the paddy field at CNRRI were sampled and immediately frozen in liquid nitrogen. The activities of reactive oxygen species (ROS) scavenging enzymes, including peroxidase (POD), superoxide dismutase (SOD), and catalase (CAT), as well as the contents of malonaldehyde (MDA) and soluble proteins (SP), were determined following the manufacturer’s instructions (Nanjing Jiancheng Bioengineering Institute, Nanjing, China). The flag leaves from WT and *psl85* at the heading stage were used for measuring photosynthetic parameters, including the net photosynthetic rate (*Pn*, μmol m^−2^ s^−1^), stomatal conductance (*Gs*, mmol m^−2^ s^−1^), intercellular CO_2_ concentration (*Ci*, μmol mol^−1^), and transpiration rate (*Tr*, mmol·m^−2^ s^−1^). The measurements were conducted at 9:00–10:00 under field conditions at CNRRI using a portable L-6400XT (LI-COR, Lincoln, NB, USA), with photosynthetic photon flux density (PPFD) of 1500 μmol m^−2^ s^−1^ and reference CO_2_ of 400 µmol·mol^−1^ in the cuvette. The means from three replicates in all experiments were used for analysis.

### 4.5. TUNEL Assay

The TUNEL assay was performed by using a Fluorescein in Situ Cell Death Detection Kit following the manufacturer’s instructions (Roche, Basel, Switzerland). The methods used for sectioning and fluorescence labeling were as previously reported [6].

### 4.6. Transmission Electron Microscopy and Paraffin Section

Fully expanded flag leaves of WT and *psl85* plants grown under field conditions at the heading stage were collected to perform transmission electron microscopy (TEM), as described previously [14]. Samples were examined with a Tecnai G2F20 transmission electron microscope at the College of Agriculture and Biotechnology, Zhejiang University. The third internodes of the main stem in WT and *psl85* at the mature stage were used for paraffin sections, as described previously [48].

### 4.7. Darkness- and ABA-Induced Leaf Senescence

Fully expanded leaves from WT and *psl85* plants at the tillering stage were cut into ~2 cm pieces, and then floated on 10 mL water or 50 μM ABA solution in Petri dishes. The samples were incubated at 30 °C in darkness or continuous light for five days.

### 4.8. ABA Level Analysis

Extraction and determination of ABA level from the top first leaves at different growth stages was performed as previously described [6]. Phytodetek ABA enzyme-linked immunosorbent assay (ELISA) Kit (Agdia, Inc., Elkhart, IN, USA) was used for quantitative analysis of ABA. The means from three measurements were used for analysis.

### 4.9. Gene Expression Analysis

Total RNA was extracted using a NucleoZOL Reagent Kit (MACHEREY-NAGEL, Düren, Germany) according to the manufacturer’s instructions. RNA was reverse-transcribed using the ReverTra Ace qPCR RT Master Mix with genomic DNA (gDNA) Remover Kit (Toyobo, Osaka, Japan). Real-time fluorescent quantitative PCR (qRT-PCR) was carried out using the FastStar Essential DNA Green Master Kit (Roche, Basel, Switzerland) and performed on a Thermal Cycle Dice Real Time System (Takara, Kusatsu, Japan). Rice ubiquitin (*LOC_Os03g13170*) was used as an internal control. The primers used for qRT-PCR are listed in Table S2. The means from three replicates were used for analysis.

### 4.10. SDS-PAGE and Immunoblot Analysis

Total proteins were extracted from fully expanded flag leaves of WT and *psl85* plants at the heading stage. Leaf tissues (100 mg) were grounded in liquid nitrogen and placed into 600 μL extraction buffer (0.4 mM Tris-HCl, pH 7.5, 5 mM NaCl, 6.25 μM MgCl_2_, 10 μM EDTA, 10 μM dl-dithiothreitol, 1% Triton X-100, 2% protease inhibitor) in a tube, then incubated in a shaker at 80 rpm, 4 °C for 30 min. Homogenates were centrifuged at 4 °C with 10,000· *g* for 20 min, and the total supernatant proteins of WT and *psl85* were quantified into the same concentration by a BCA Protein Assay Kit (TIANGEN, Beijing, China). The quantified total proteins were denatured at 95 °C for 5 min. Total proteins (10 μL) of WT and *psl85* were subjected to 12% (*w*/*v*) polyacrylamide sodium dodecyl sulfate-polyacrylamide gel electrophoresis (SDS-PAGE) and the resolved proteins were transferred onto a nitrocellulose membrane. Antibodies against the photosystem proteins Lhca1, Lhca2, Lhcb2, and PsbD (Agrisera, Vännäs, Sweden) were used for Western blot analysis. The photosystem protein levels were detected using an ECL Prime Western Blotting System (GE Healthcare, Chicago, IL, USA) according to the manufacturer’s protocol.

### 4.11. Genetic Analysis and Gene Mapping 

The F_1_ plants from the cross between *psl85*/Moroberekan were grown in the paddy field at CNRRI for determining dominance or recessiveness of *PSL85*, and the selfed F_2_ individual plants were used for segregation analysis. Equal amounts of leaf blades from each of ten WT plants and ten *psl85* plants were collected for DNA extraction to form a WT DNA pool and a *psl85* type DNA pool, respectively. These two pools were subjected to preliminary linkage analysis of the mutation by genotyping 362 polymorphic simple sequence repeat (SSR) markers covering 12 chromosomes. Subsequently, 352 *psl85* type individual plants from the F_2_ population were genotyped to determine the physical location of *PSL85*.

## Figures and Tables

**Table 1:** Function annotation of the candidate genes.

ORF	Gene ID	Annotation
ORF1	LOC_Os07g10610	Expressed protein
ORF2	LOC_Os07g10620	Expressed protein
ORF3	LOC_Os07g10630	Expressed protein
ORF4	LOC_Os07g10640	Hypothetical protein
ORF5	LOC_Os07g10650	Hypothetical protein
ORF6	LOC_Os07g10660	Ribosomal protein, putative, expressed
ORF7	LOC_Os07g10670	Expressed protein
ORF8	LOC_Os07g10680	Polygalacturonase, putative, expressed

## Supplementary Materials

Supplementary materials can be found at http://www.mdpi.com/1422-0067/19/8/2339/s1.

## References

[1] Quirino B.F., Noh Y.S., Himelblau E., Amasino R.M. (2000). Molecular aspects of leaf senescence. Trends Plant Sci..

[2] Buchanan-Wollaston V., Page T., Harrison E., Breeze E., Lim P.O., Nam H.G., Lin J.F., Wu S.H., Swidzinski J., Ishizaki K. (2005). Comparative transcriptome analysis reveals significant differences in gene expression and signaling pathways between developmental and dark/starvation-induced senescence in *Arabidopsis*. Plant J..

[3] Woo H.R., Chung K.M., Park J.H., Oh S.A., Ahn T., Hong S.H., Jang S.K., Nam H.G. (2001). ORE9, an F-box protein that regulates leaf senescence in *Arabidopsis*. Plant Cell.

[4] Kim H.J., Ryu H., Hong S.H., Woo H.R., Lim P.O., Lee I.C., Sheen J., Nam H.G., Hwang I. (2006). Cytokinin-mediated control of leaf longevity by AHK3 through phosphorylation of ARR2 in *Arabidopsis*. Proc. Natl. Acad. Sci. USA.

[5] Yang S.D., Seo P.J., Yoon H.K., Park C.M. (2011). The *Arabidopsis* NAC transcription factor VNI2 integrates abscisic acid signals into leaf senescence via the *COR/RD* genes. Plant Cell.

[6] He Y., Li L., Zhang Z., Wu J.L. (2018). Identification and comparative analysis of premature senescence leaf mutants in rice (*Oryza sativa* L.). Int. J. Mol. Sci..

[7] Woo H.R., Masclaux-Daubresse C., Lim P.O. (2018). Plant senescence: How plants know when and how to die. J. Exp. Bot..

[8] Kirchhoff H., Tremmel I., Haase W., Kubitscheck U. (2004). Supramolecular photosystem II organization in grana thylakoid membranes: Evidence for a structured arrangement. Biochemistry.

[9] Schelbert S., Aubry S., Burla B., Agne B., Kessler F., Krupinska K., Hörtensteiner S. (2009). Pheophytin pheophorbide hydrolase (pheophytinase) is involved in chlorophyll breakdown during leaf senescence in *Arabidopsis*. Plant Cell..

[10] Lee S.H., Sakuraba Y., Lee T., Kim K.W., An G., Lee H.Y., Paek N.C. (2015). Mutation of *Oryza sativa CORONATINE INSENSITIVE 1b* (*OsCOI1b*) delays leaf senescence. J. Integr. Plant Biol..

[11] Sakuraba Y., Han S.H., Yang H.J., Piao W., Paek N.C. (2016). Mutation of *Rice Early Flowering3.1* (*OsELF3.1*) delays leaf senescence in rice. Plant Mol. Biol..

[12] Roy N., Bagchi S., Raychaudhuri P. (2012). Damaged DNA binding protein 2 in reactive oxygen species (ROS) regulation and premature senescence. Int. J. Mol. Sci..

[13] Yang L., Ye C., Zhao Y., Cheng X., Wang Y., Jiang Y.Q., Yang B. (2018). An oilseed rape WRKY-type transcription factor regulates ROS accumulation and leaf senescence in *Nicotiana benthamiana* and *Arabidopsis* through modulating transcription of *RbohD* and *RbohF*. Planta.

[14] Huang Q.N., Shi Y.F., Zhang X.B., Song L.X., Feng B.H., Wang H.M., Xu X., Li X.H., Guo D., Wu J.L. (2016). Single base substitution in *OsCDC48* is responsible for premature senescence and death phenotype in rice. J. Integr. Plant Biol..

[15] Zhang X.B., Feng B.H., Wang H.M., Xu X., Shi Y.F., He Y., Chen Z., Sathe A.P., Shi L., Wu J.L. (2018). A substitution mutation in *OsPELOTA* confers bacterial blight resistance by activating the salicylic acid pathway. J. Integr. Plant Biol..

[16] Wu L., Ren D., Hu S., Li G., Dong G., Jiang L., Hu X., Ye W., Cui Y., Zhu L. (2016). Down-regulation of a nicotinate phosphoribosyltransferase gene, *OsNaPRT1*, leads to withered leaf tips. Plant Physiol..

[17] Buchanan-Wollaston V., Earl S., Harrison E., Mathas E., Navabpour S., Page T., Pink D. (2003). The molecular analysis of leaf senescence–a genomics approach. Plant Biotechnol. J..

[18] Lim P.O., Kim H.J., Nam H.G. (2007). Leaf senescence. Annu. Rev. Plant Biol..

[19] Zhao Y., Chan Z., Gao J., Xing L., Cao M., Yu C., Hu Y., You J., Shi H., Zhu Y. (2016). ABA receptor PYL9 promotes drought resistance and leaf senescence. Proc. Natl. Acad. Sci. USA.

[20] Liang C., Wang Y., Zhu Y., Tang J., Hu B., Liu L., Ou S., Wu H., Sun X., Chu J. (2014). OsNAP connects abscisic acid and leaf senescence by fine-tuning abscisic acid biosynthesis and directly targeting senescence-associated genes in rice. Proc. Natl. Acad. Sci. USA.

[21] Gao S., Gao J., Zhu X., Song Y., Li Z., Ren G., Zhou X., Kuai B. (2016). ABF2, ABF3, and ABF4 promote ABA mediated chlorophyll degradation and leaf senescence by transcriptional activation of chlorophyll catabolic genes and senescence-associated genes in *Arabidopsis*. Mol. Plant.

[22] Mao C., Lu S., Lv B., Zhang B., Shen J., He J., Luo L., Xi D., Chen X., Ming F. (2017). A rice NAC transcription factor promotes leaf senescence via ABA biosynthesis. Plant Physiol..

[23] Kong Z., Li M., Yang W., Xu W., Xue Y. (2006). A novel nuclear-localized CCCH-type zinc finger protein, OsDOS, is involved in delaying leaf senescence in rice. Plant Physiol..

[24] Jiao B.B., Wang J.J., Zhu X.D., Zeng L.J., Li Q., He Z.H. (2012). A novel protein RLS1 with NB-ARM domains is involved in chloroplast degradation during leaf senescence in rice. Mol. Plant.

[25] Piao W., Kim E.Y., Han S.H., Sakuraba Y., Paek N.C. (2015). Rice phytochrome B (OsPhyB) negatively regulates dark- and starvation-induced leaf senescence. Plants.

[26] Jing H.C., Nam H.G. (2012). Leaf senescence in plants: From model plants to crops, still so many unknowns. J. Integr. Plant Biol..

[27] Xu H., Zhang L., Li R., Wang X., Liu S., Liu X., Jing Y., Xiao J. (2018). SKL1 is essential for chloroplast development in *Arabidopsis*. Front. Plant Sci..

[28] Schlimme M., Blaschke L., Lagrimini L.M., Polle A. (2002). Growth performance and lignification in tobacco with suppressed apoplastic anionic peroxidase activity under ambient and elevated CO_2_ concentrations. Int. J. Plant Sci..

[29] Breusegem F.V., Dat J.F. (2006). Reactive oxygen species in plant cell death. Plant Physiol..

[30] Petrov V., Hille J., Mueller-Roeber B., Gechev T.S. (2015). ROS-mediated abiotic stress-induced programmed cell death in plants. Front. Plant Sci..

[31] Mittova V., Guy M., Tal M., Volokita M. (2004). Salinity up-regulates the antioxidative system in root mitochondria and peroxisomes of the wild salt tolerant tomato species *Lycopersicon pennellii*. J. Exp. Bot..

[32] Affenzeller M.J., Darehshouri A., Andosch A., Lütz C., Lütz-Meindl U. (2009). Salt stress-induced cell death in the unicellular green alga *Micrasterias denticulata*. J. Exp. Bot..

[33] Leng Y., Yang Y., Ren D., Huang L., Dai L., Wang Y., Chen L., Tu Z., Gao Y., Li X. (2017). A rice *PECTATE LYASE-LIKE* gene is required for plant growth and leaf senescence. Plant Physiol..

[34] Liu X., Li Y. (2016). Varietal difference in the correlation between leaf nitrogen content and photosynthesis in rice (*Oryza sativa* L.) plants is related to specific leaf weight. J. Integr. Agric..

[35] Ma X., Sun X., Li C., Huan R., Sun C., Wang Y., Xiao F., Wang Q., Chen P., Ma F. (2017). Map-based cloning and characterization of the novel yellow-green leaf gene *ys83* in rice (*Oryza sativa*). Plant Physiol. Biochem..

[36] Cutler S.R., Rodriguez P.L., Finkelstein R.R., Abrams S.R. (2010). Abscisic acid: emergence of a core signaling network. Annu. Rev. Plant Biol..

[37] Schwartz S.H., Qin X., Zeevaart J.A. (2003). Elucidation of the indirect pathway of abscisic acid biosynthesis by mutants, genes, and enzymes. Plant Physiol..

[38] Nambara E., Marion-Poll A. (2005). Abscisic acid biosynthesis and catabolism. Annu. Rev. Plant Biol..

[39] Kushiro T., Okamoto M., Nakabayashi K., Yamagishi K., Kitamura S., Asami T., Hirai N., Koshiba T., Kamiya Y., Nambara E. (2004). The *Arabidopsis* cytochrome P450 CYP707A encodes ABA 8′-hydroxylases: Key enzymes in ABA catabolism. EMBO J..

[40] Saito S., Hirai N., Matsumoto C., Ohigashi H., Ohta D., Sakata K., Mizutani M. (2004). *Arabidopsis* CYP707As encode (+)-abscisic acid 8′-hydroxylase, a key enzyme in the oxidative catabolism of abscisic acid. Plant Physiol..

[41] Deng L.C., Qin P., Liu Z., Wang G., Chen W., Tong J., Xiao L., Tu B., Sun Y., Yan W. (2017). Characterization and fine-mapping of a novel premature leaf senescence mutant *yellow leaf and dwarf 1* in rice. Plant Physiol. Biochem..

[42] Yu H., Ruan B., Wang Z., Ren D., Zhang Y., Leng Y., Zeng D., Hu J., Zhang G., Zhu L. (2017). Fine mapping of a novel *defective glume 1* (*dg1*) mutant, which affects vegetative and spikelet development in rice. Front. Plant Sci..

[43] Jiang H., Li M., Liang N., Yan H., Wei Y., Xu X., Liu J., Xu Z., Chen F., Wu G. (2007). Molecular cloning and function analysis of the *stay green* gene in rice. Plant J..

[44] Tang Y., Li M., Chen Y., Wu P., Wu G., Jiang H. (2011). Knockdown of Os*PAO* and Os*RCCR1* cause different plant death phenotypes in rice. J. Plant Physiol..

[45] Lee R.H., Wang C.H., Huang L.T., Chen S.C. (2001). Leaf senescence in rice plants: cloning and characterization of senescence up-regulated genes. J. Exp. Bot..

[46] Wang X., Yan Y., Li Y., Chu X., Wu C., Guo X. (2014). *GhWRKY40*, a multiple stress-responsive cotton WRKY Gene, plays an important role in the wounding response and enhances susceptibility to *Ralstonia solanacearum* infection in transgenic *Nicotiana benthamiana*. PLoS ONE.

[47] Yin Z., Chen J., Zeng L., Goh M., Leung H., Khush G.S., Wang G.L. (2000). Characterizing rice lesion mimic mutants and identifying a mutant with broad-spectrum resistance to rice blast and bacterial blight. Mol. Plant Microbe Interact..

[48] He Y., Shi Y.F., Zhang X.B., Wang H.M., Xu X., Wu J.L. (2017). Identification of a gravitropism-deficient mutant in rice. Rice Sci..

