# Supplementary material for: Genetic and Physio-Biochemical Characterization of a Novel Premature Senescence Leaf Mutant in Rice (Oryza sativa L.)
Source: Int J Mol Sci. 2018 Aug 9;19(8):2339. doi: 10.3390/ijms19082339 (PMC6122088; doi:10.3390/ijms19082339)
Supplement: Supplementary file 1 [file ijms-19-02339-s001.pdf]

Table S1. Agronomic traits of WT and *psl85*.

Material	Seed-Setting (%)	No. Unfilled Grains	No. Filled Grains	1,000-Grain Weight (g)	No. Panicle	Panicle Length (cm)	Plant Height (cm)
WT	85.99 ± 0.86	322 ± 46.29	1995 ± 417.25	22.01 ± 0.1	14.67 ± 0.58	26.6 ± 0.53	101.67 ± 2.87
psl85	56.47 ± 3.55**	310 ± 80.73	395 ± 52.43**	22.48 ± 0.65	14.67 ± 1.53	21.47 ± 1.01**	80.17 ± 1.89**

Values are means ± SD (n = 3), ** indicates significance at $p \leq 0.01$ by Student's t-test.

Table S2. The primers used in this study.

Primers	Forward Sequence (5′-3′)	Reverse Sequence (5′-3′)	Accession Number
RM5752	TTGCAATTAATTCGATCTCC	GCAGATCGATTCGTTAGTTC	
RM8262	AACAGATATACTCGGGCAGCATTAGC	TGACTCCTCCGTGGTAAACACC	
RM1186	ATATGGTCATTGGCTGGGAAAGAGG	CACCATTATCCTGCGGGTAGGC	
RM8247	TCACCCTTCTTGTCAAGCAAACC	GAGGATCAGGATAGAACATGAGATGC	
RM21183	TTCTCTCGTGAGGAGGAGACAGG	TCCTCCTTCCAACCACCTTTCC	
Ubiquitin	CCCTCCACCTCGTCCTCAG	AGATAACAACGGAAGCATAAAAGTC	*LOC_Os03g13170*
ORF1	GTCATCGACCTCATCGGGGA	ACAGGGATGTCGAAACGGAA	*LOC_Os07g10610*
ORF2	TCATGGCTAGAGTGTTCCTGC	CCATTACCATCGGCGGTTTG	*LOC_Os07g10620*
ORF3	CAAACCTTGTTCGTCCCATT	GCTGAAGCAAGGTTCCATTC	*LOC_Os07g10630*
ORF4	TTGTGCCGTCGCCAGACTCT	GCAGCTTGCCACCATCATT	*LOC_Os07g10640*
ORF5	CATGCAGGGTCCTTATGTA	CACCCCTTCATTATCGCC	*LOC_Os07g10650*
ORF6	GGCCACCTTCGATTGTCTCA	GTCCGTGTACTCCTGGAACG	*LOC_Os07g10660*
ORF7	CACGACGACCTCTCCTTGAT	TGCGCTGTATGTCATCACAA	*LOC_Os07g10670*
ORF8	ACAACCAACGGAGCTCGCA	ACAACCAACGGAGCTCGCA	*LOC_Os07g10680*
CAO	TTGGCACAAATGGAGACCC	GCTGCACTGGACCAGACAC	*LOC_Os10g41780*
rbcS	CCGTGAGAACCACAGATCCC	ACGTTGTCGAAGCCGATGAT	*LOC_Os12g17600*
rbcL	ATCGTGCTCGCGGTATCTTT	ACCAGGTGCATTACCCCAAG	*LOC_Os12g10580*
HEMA1	ACACGCCATCTGTTTGAGGT	CAAGCCTCCACTGTTTTGCC	*LOC_Os10g35840*
psbA	TGTAGCTGGTGTATTCGGCG	ATAACCATGAGCGGCCACAA	plastogene
porA	ATCACCAAGGGCTACGTCTC	GAGTTGTTGTTCCAGCTCCA	*LOC_Os04g58200*
NPH1a	CACTTGCAACCAATGCGTGA	ATCCGGGAGTTCCTTTGCAG	*LOC_Os11g01140*
CHLI	CGGAGTAACCTTGGTGCTGT	CTTGGCAGCCCTGTTAGTCA	*LOC_Os03g36540*
psbS	CTGAGCCGAAGCCAAAGTTC	ATCCCCGTCTCCAGGTTCAG	*LOC_Os01g64960*
CHLH	TGACTCAGACCCGACAAAGC	TCCCCTCGTACCACTTAGGG	*LOC_Os03g20700*
cab2R	GTTCTCCATGTTCGGCTTCT	GACGAAGTTGGTGGCGTAG	*LOC_Os01g41710*
SGR	AGGGGTGGTACAACAAGCTG	GCTCCTTGCGGAAGATGTAG	*LOC_Os09g36200*
RCCR1	CGCATTTCCTCATGGAATTT	CTTCTCACGCTGTTTGTCCA	*LOC_Os10g25030*
Osh36	AACGCATTTGTGGTTGGCTC	TCAACTTTGGCCGGTGTCTT	*LOC_Os05g39770*
OsI57	ACCCTAAAGTAAATGAAGTC	CCTGCTCTTGTCTTGTTA	*LOC_Os02g57260*
OsNCED1	ACCATGAAGTCCATGAGGCT	TCTCGTAGTCTTGGTCTTGG	*LOC_Os02g47510*
OsNCED	CAAGTTCGAGTACGGCGAGG	GACAGGATGTAGCCGTCGTC	*LOC_Os03g44380*

3			
OsNCED 4	TCGGGAGGTACGACTTCCAT	TTGAGGTACGGCTTGGACAC	*LOC_Os07g05940*
OsZEP	GGATGCCATTGAGTTTGGTT	TGGCTGACTGAAGTCTCTCG	*LOC_Os04g37619*
OsABA8ox1	CAAGCCCAACACGTTCATGC	TGTACTTGGTTGCGAGGTGG	*LOC_Os02g47470*
OsABA8ox2	CTACTGCTGATGGTGGCTGA	CCCATGGCCTTTGCTTTAT	*LOC_Os08g36860*
OsABA8ox3	AGTACAGCCCATTCCCTGTG	ACGCCTAATCAAACCATTGC	*LOC_Os09g28390*
